# High and Distinct Range-Edge Genetic Diversity despite Local Bottlenecks

**DOI:** 10.1371/journal.pone.0068646

**Published:** 2013-07-10

**Authors:** Jorge Assis, Nelson Castilho Coelho, Filipe Alberto, Myriam Valero, Pete Raimondi, Dan Reed, Ester Alvares Serrão

**Affiliations:** 1 Centro de Ciências do Mar do Algarve, CIMAR-Laboratório Associado, University of Algarve, Campus de Gambelas, Faro, Portugal; 2 Department of Biological Sciences, University of Wisconsin-Milwaukee, Milwaukee, Wisconsin, United States of America; 3 Centre National de la Recherche Scientifique Université́ Pierre et Marie Curie, UMR CNRS, UPMC 7144, Roscoff, France; 4 Department of Biology, University of California Santa Cruz, Santa Cruz, California, United States of America; 5 Marine Science Institute, University of California Santa Barbara, Santa Bárbara, California, United States of America; University of Canterbury, New Zealand

## Abstract

The genetic consequences of living on the edge of distributional ranges have been the subject of a largely unresolved debate. Populations occurring along persistent low latitude ranges (rear-edge) are expected to retain high and unique genetic diversity. In contrast, currently less favourable environmental conditions limiting population size at such range-edges may have caused genetic erosion that prevails over past historical effects, with potential consequences on reducing future adaptive capacity. The present study provides an empirical test of whether population declines towards a peripheral range might be reflected on decreasing diversity and increasing population isolation and differentiation. We compare population genetic differentiation and diversity with trends in abundance along a latitudinal gradient towards the peripheral distribution range of *Saccorhiza* *polyschides* , a large brown seaweed that is the main structural species of kelp forests in SW Europe. Signatures of recent bottleneck events were also evaluated to determine whether the recently recorded distributional shifts had a negative influence on effective population size. Our findings show decreasing population density and increasing spatial fragmentation and local extinctions towards the southern edge. Genetic data revealed two well supported groups with a central contact zone. As predicted, higher differentiation and signs of bottlenecks were found at the southern edge region. However, a decrease in genetic diversity associated with this pattern was not verified. Surprisingly, genetic diversity increased towards the edge despite bottlenecks and much lower densities, suggesting that extinctions and recolonizations have not strongly reduced diversity or that diversity might have been even higher there in the past, a process of shifting genetic baselines.

## Introduction

Understanding the processes shaping genetic diversity of range-edge populations is an important current challenge, particularly where rich former glacial refugia populations with high conservation value have become isolated in decreasing suitable habitat islands [1]. Accordingly, empirical data for populations at distributional edges do not all support the same general geographic pattern. These can vary from diverse persistent populations where habitat has remained favourable over the long term [2] to margins with small and low density populations where genetic diversity may be lower and clonal reproduction and inbreeding may prevail [3,4]. Such populations might represent the last refugia of threatened distinct genetic diversity [5,6].

The genetic diversity of a population reflects both current and past events. The prediction of lower genetic diversity as a response to reductions in effective population size and gene flow towards edges [7,8] assumes a current trend in abundance, from abundant central regions of distribution towards small and less dense populations; an assumption that has rarely been confirmed empirically. Many studies failed to find evidence for larger abundances at the centre of species distributions [9] and the few that supported the hypothesis were limited to a small number of species [10] and sites [11,12]. Yet, in most studies considering genetic diversity, a decrease in within population diversity and an increase in genetic differentiation between populations were observed towards the peripheral range [8]. The prevalence of effects of current population abundance patterns over past history in determining current genetic diversity might reflect the fact that extinction is forever, even when caused by unsuitable conditions that are temporary. Once lost, unique alleles occurring at range edges cannot reappear no matter how favourable the habitat becomes. The loss of adaptive variation towards range edges may compromise a population’s ability to evolve [13,14], thereby increasing the threat of extinction [15–17]. This might be accentuated in isolated populations of annual species, which are naturally more prone to local bottlenecks and extinctions [18]. Thus, areas where past history created higher genetic diversity due to long term persistence of populations exposed to climatic refugia, or gene flow from differentiated populations [7,19,20] are expected to be lost by current bottlenecks, although regional diversity might retain a diverse signature [5].

The relationship between the geographic distributions of abundance and genetic diversity now appear more complex and interesting than previously assumed. This complexity strongly alters simplistic biogeographic predictions about population dynamics [11], genetic structure of populations, and species responses to climate change [21]. To move beyond simplistic assumptions it is necessary to integrate more sources of data (e.g., population demography and genetic structure) to narrow the range of viable hypotheses that explain the ecological and evolutionary mechanisms underlying species distribution [22]. Moreover, studies frequently compare samples from sites with high abundances of focal species with very few in the peripheral range, assuming less abundance and higher levels of isolation without an empirical verification of demographic variables [23,24]. This approach is unlikely to distinguish whether geographic variation in genetic structure covaries with contemporary population abundance and peripheral isolation, or is the result of historical processes [7].

An interesting model to study the genetic implications of distributional ranges from an abundant to a peripheral region is the Portuguese coast along western Iberia. This is a region with a biogeographical interface where a wide range of marine species show latitudinal clines in abundance, along a narrow strip of shoreline habitat essentially in one dimension, from North to South [25,26]. One such species is the annual kelp *Saccorhiza* *polyschides* , which is the main canopy species forming kelp forests in this region. This species sharply declines from being a highly abundant dominant species in the north to being rare near its distributional limit in the south. Such a spatially unidimensional model has previously proven effective in testing phylogeographic hypotheses in marine species [9,27].

This study addresses the genetic consequences of a sharp decline in abundance at the distributional margin of *S* *. polyschides* . This was achieved by quantifying the latitudinal gradient in population density towards the southern edge of distribution, and assessing whether it was related to decreasing genetic diversity and increasing differentiation. We used this information to test whether populations closer to the range boundary show no change in (1) relative densities, (2) fragmentation (3), genetic differentiation (4), genetic diversity and (5) signatures of recent bottleneck events (i.e. population turnover)

## Methods

### Ethics Statement

No specific permits were required for the sampling as the sites were not privately-owned or protected in any way, and the field studies did not involve endangered or protected species.

### Focal species, study area and sample collection

The annual kelp *S* *. polyschides* is an important ecosystem-building species in European waters [28]. This short-lived pioneer species is distributed from the western coast of Norway, extending southward to Scotland, Ireland, Wales, southwest England, Brittany, France and along the Spanish and Portuguese coasts, meeting its southern boundary in Morocco. It can also be found in few deep (~ 30m) isolated sites of the Western Mediterranean Sea [29].

Sampling sites for *S* *. polyschides* covered the entire west coast of Portugal, including searches in areas beyond the current southern limit of the species in mainland. This region, which happens to coincide with the boundaries of a political country, is an excellent model coastline to study the genetic implications of distributional ranges from an abundant to a peripheral region for 3 main reasons: 1) Gradual abundance gradient: It coincides perfectly with a sharp linear gradient from abundant continuous populations in the North, to small patchy fragmented populations in the center-southwest, to complete absence of the species along the southern coast. 2) Availability of long-term historical records of the species occurrence (particularly from Assis et al., 2009) showing recent range shifts along this coast. 3) Coastal southern limit: The southwest of Portugal is the southern range edge of the coastal distribution of S. polyschides. Beyond this region there are only 2 areas that support S. polyschides. Both are separated by hundreds to thousands of km in opposite directions (southwards, eastwards and westwards), are not part of the coastal distribution and thus are not useful to address the question of this paper. These two isolated areas are a) the strong upwelling points of Alboran (East) and Morocco (South), and b) the very deep offshore banks (e.g. Gorringe and Messina) where oceanic waters are so transparent that they allow the species to occur at depth ranges of about 40-80 m, much beyond the coastal depth ranges. Species distributions are not always linear with latitude and pockets or islands can occur beyond the limits of the linear latitudinal distribution, due to particular unique combinations of habitat conditions [6,30].

Along this sampled area populations recruit in spring and reach their highest abundance during summer. Adult individuals (sporophytes) die in the autumn and are absent during the winter, starting to recruit again in spring [31].

**Figure 1:**
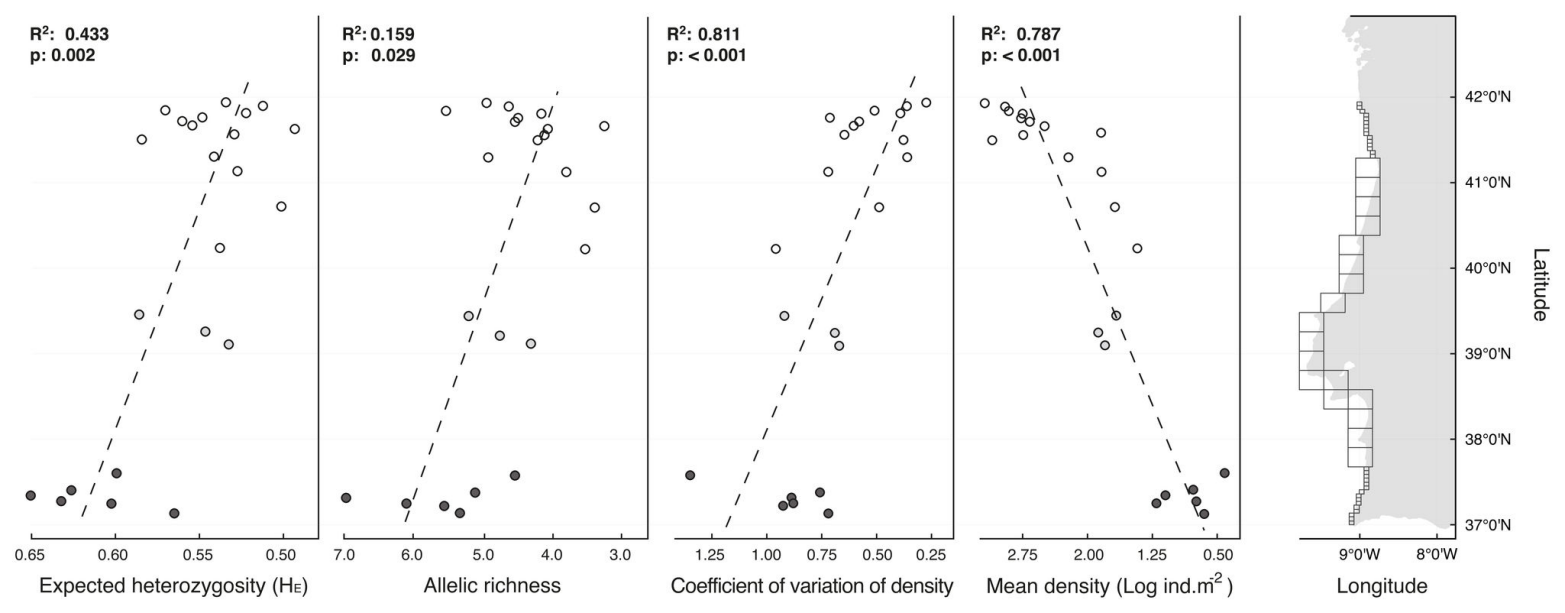
Sampling scheme for *S* *. polyschides* (cells represent the sampling units) within 25 km cells along the study range, and for detailed northern-southern comparisons within sub-cells of 5 km. Expected heterozygosity (H_E_), allele number, coefficient of variation of density and mean density (values increase from right to left; North: white circles, Center: grey circles, South: black circles) plotted against latitude (decimal degrees at WGS84). R-squared and p-values for linear models (dashed lines) fitted for sites with density records and genetic samples.

The Portuguese coastline was divided into 25 juxtaposed cells of 25 km from 42,0^°^ N to 37,0^°^ N, and for better resolution in North–South comparisons, the sampling effort was intensified at the 3 northernmost and 3 southernmost cells by dividing the 25 km cells into 5 sub-cells of 5 km (Figure 1). Forests of *S* *. polyschides* were sampled at the centroid sites of each cell, during the summers of 2008 and 2010, by means of SCUBA diving and snorkelling. If no kelp was found, at least two more randomly chosen sites in the same cell were surveyed with the same objective.

All sampling was conducted at comparable depths, shallower than 8 m, in order to avoid confounding latitude with the effects of depth, since the abundance of *S* *. polyschides* varies with depth [32]. Species distribution was assessed in 2008 and 2010, by collecting presence and absence records at each sampling site. In the summer of 2010, the density of *S* *. polyschides* was also sampled and tissue was collected for genetic analysis. For density estimates, four quadrats (0.5 m x 0.5 m) were placed along three 20 m long transects haphazardly laid in an extant kelp forest, at 5, 10, 15 and 20 m (totalling 12 quadrats). In each quadrat all *S* *. polyschides* individuals were counted. For genetic analyses, 30 individuals were sampled along transects by removing a piece of the blade above the meristem. These were preserved in silica drying crystals until DNA extraction.

### Population distribution and spatial heterogeneity

To evaluate inter-annual variability in the distribution and abundance of *S* *. polyschides* , the presence and absence records were plotted for both sampling years, together with an extensive list of historical geo-referenced occurrences gathered from literature (dataset and references can be obtained from the authors upon request). Furthermore, mean density of *S* *. polyschides* per site (expressed as individuals per m^2^) was calculated with the quadrat counts for 2010 samples. To infer the fragmentation level of kelp per site, the coefficient of variation among all quadrat counts was determined as a measure of dispersion that represents within site landscape spatial heterogeneity [33] independently of the mean density. To test whether *S* *. polyschides* was less dense or more heterogeneous at sites towards the distributional edge, linear regression models were fitted between latitude and density, and between latitude and the coefficient of variation of density. Predictors were transformed if needed (log), homogeneity of variances and normality of models were assessed by graphical inspection of the residuals versus fitted values [34] and by performing the Shapiro-Wilk test [35] with H_0_: the residuals were normally distributed.

### Microsatellite amplification, scoring and correction

Genomic DNA was isolated from 5 to 10 mg of dried tissue using a CTAB method and Filter Plates (MSFBN6B10, Milllipore) as described in [36]. A total of seven microsatellite loci (2F7, 1A1(2), 1E10, 3A10, 2A4, 3D12 and 2B3 [37]) were amplified for all sampling units. PCR reactions in 15 µl contained ±20 ng of DNA, 0.16 µM of forward 5’ fluorochrome labeled primer and 0.33 µM of reverse primer, 0.8 mM of dNTPs (Bioline), 2.0 or 2.5 mM of MgCl_2_, 3.0 µl of 5x PCR Buffer and 0.4 U of GoTaq Polymerase (Promega, Madison, WI). Cycling conditions consisted of an initial denaturing step of 5 min at 95^°^C, followed by 35 cycles of 30 s at 95^°^C, 30 s at annealing temperature, 45 s at 72^°^C, and a final elongation step at 72^°^C for 20 minutes. All PCR reactions were performed on a GeneAmp 9700 thermocycler (PE Applied Biosystems, Foster City, California, USA). Fragment length was analyzed on an ABI PRISM 3130xl DNA analyzer (Applied Biosystems) using the GeneScan 500 LIZ standard.

Raw allele sizes were scored using the software STRand [38] and binned into allele classes using the MsatAllele package [39] in the R software [40]. Loci were tested for null alleles and scoring errors using the software Microchecker [41]. Deviations from Hardy–Weinberg equilibrium and for linkage disequilibrium between pairs of loci were computed with FSTAT [42].

### Estimates of genetic diversity

Genetic diversity, as allelic richness (A) and Nei’s gene diversity (expected heterozygosity; H_E_), were determined per locus and per site for all loci, using FSTAT. To test whether genetic diversity decreased towards the edge, a linear regression model was fitted between latitude and genetic diversity per site (A and H_E_). Homogeneity and normality of both models was assessed. Allelic richness was also computed for each genetic cluster (see below), standardised to the number of individuals and coastal distance range of the smallest cluster, using StandArich [43]. The number of unique alleles per genetic cluster was also determined.

### Population genetic structure

The number of distinct genetic clusters (K) present in the studied region was inferred by running software Structure [44] with a burning time of 2x10^5^ repetitions and 1x10^6^ iterations exploring K from 1 to 8, with admixture allowed and without any a priori population assignments. The estimation of the likely number of clusters used the log probability of data Pr(X/*K*) [44] for each value of K and the DK criteria of [45]. For the most likely K, population assignment was graphically displayed with Distruct [46]. The patterns of genetic differentiation were illustrated through a Factorial Correspondence Analysis (FCA) of population multiscores computed using GENETIX 4.05 [47]. Moreover, the association between the mean genetic similarity calculated over all loci and the geographic regions was shown by a consensus neighbour-joining (NJ) network based on Cavalli-Sforza & Edwards [48] genetic distances among all sites, computed using the software Populations [49] with 1x10^5^ bootstrap resamplings.

Levels of differentiation between sites were inferred using the *F*_ST_ estimator computed over loci, and within genetic groups using both *F*_ST_ and Jost’s D [50]. Hierarchical analysis of molecular variance (AMOVA) was computed using Genodive [51], based on allele frequency information under 999 permutations [52]. Variance components were extracted for 3 hierarchical levels (1) among individuals within sites (2), among sites within genetic groups and (3) among genetic groups. Genetic groups were partitioned following the outcomes of the FCA and the Bayesian clustering analysis.

Isolation by distance (IBD) was evaluated within groups, using pairwise estimates of mean genetic distance (*F*_ST_ ⁄ (1 -*F*_ST_)) between sites, against pairwise minimum marine distances. Marine distances were computed with package gdistance for R [40] with least-cost distance between sites using land mass as an infinite resistance surface. The null hypothesis of no correlation between pairwise geographic distance and genetic distance matrices [53,54] was tested using Mantel non-parametric test [55] based on 1x10^5^ permutations as implemented in Genodive.

### Inference of population bottleneck

For each sampling site, evidence for recent bottleneck events was tested using two methods: (1) heterozygosity excess [56] and (2) M-ratios [57].

Populations that have experienced a recent bottleneck are predicted to temporarily lose allelic diversity at a significantly faster rate than heterozygosity [56]. This excess in heterozygosity was tested with software Bottleneck [58] using 9999 simulations. The Two-Phase Model (TPM) was used since it’s more appropriate and realistic for microsatellites [56,58]. The frequency of step mutations was set to 0.9 (ps) and the variance of mutations to 12 (generic values, typical for many microsatellite markers [58,59];. Based on the number of loci in our dataset (less than 20), the Wilcoxon test was performed for the statistical analysis with the null hypothesis of no significant heterozygosity excess (on average) across loci [56,60].

The M-ratio test was performed with the software M_P_VAL [57]. This method is based on the premise that during a bottleneck, rare alleles are most likely to be lost, and the number of observed allelic states (k) reduces faster than the range of allele size (r), which results in a reduced M-ratio (M = k/r). Critical significance values (Mc), the lower boundary of the one-sided 95% confidence interval, were calculated using the software Critical_M [57] with 10,000 randomizations [61]. These calculations were made using ps, Dg (the size of non one-step changes) and Theta = 4*Neµ*, three parameters known to influence the Mc results [59]. Since there is no information on these parameters for the *S* *. polyschides* sampled sites, and to minimise type I errors, the Mc value for each site was calculated with the mean size of non-stepwise mutations = 3.5 and a highly conservative Theta = 10 (which assumes larger *Ne* and lower µ). The proportion of mutations was set to 0.9 as recommended by Garza & Williamson [57]. Observed M-ratios below Mc indicate a bottleneck.

## Results

### Sample collection and microsatellite amplification

Along the Portuguese coast, presence-absence records of *S* *. polyschides* were performed on 48 visited cells (Table S1). At 23 of these cells, populations were sampled for density and genetic attributes. At one particular site (#8) only 16 individuals were found, precluding accurate density estimates within quadrats although samples could still be taken for genetic analysis.

All seven loci were polymorphic across all sites (see Table S2 for the details of gene diversity, allele richness and *F*_IS_ values for each site and each locus). A total of 96 alleles were obtained from 676 genotyped individuals, ranging from 7 to 20 alleles per locus (mean = 13.71, SD = 4.39), and on a single site from 23 to 41 alleles (mean = 35.52, SD = 5.18). Significant *F*_IS_ values were obtained, particularly in southern sites (#15, 17, 19, 20, 21, 22 and 23). No linkage disequilibrium was detected between all pairs of loci (Table S2). Microchecker analyses indicated no signs of stuttering error, but with the exception of one locus (3D12), all showed evidence of null alleles, particularly 2A4 (with 0.135 ± 0.078 null alleles on average, resulting in higher Fis values compared to other loci, Table S2). Yet, null alleles were uncommon to rare across loci (null alleles per locus < 0.2 [62]), and had no consistency among sites. To account for possible null allele effects, all analyses of inter-population structure and bottleneck were run with and without locus 2A4, and its exclusion did not change the results. Hence, we did not exclude this locus from our analyses.

### Population abundance and spatial heterogeneity

**Figure 2:**
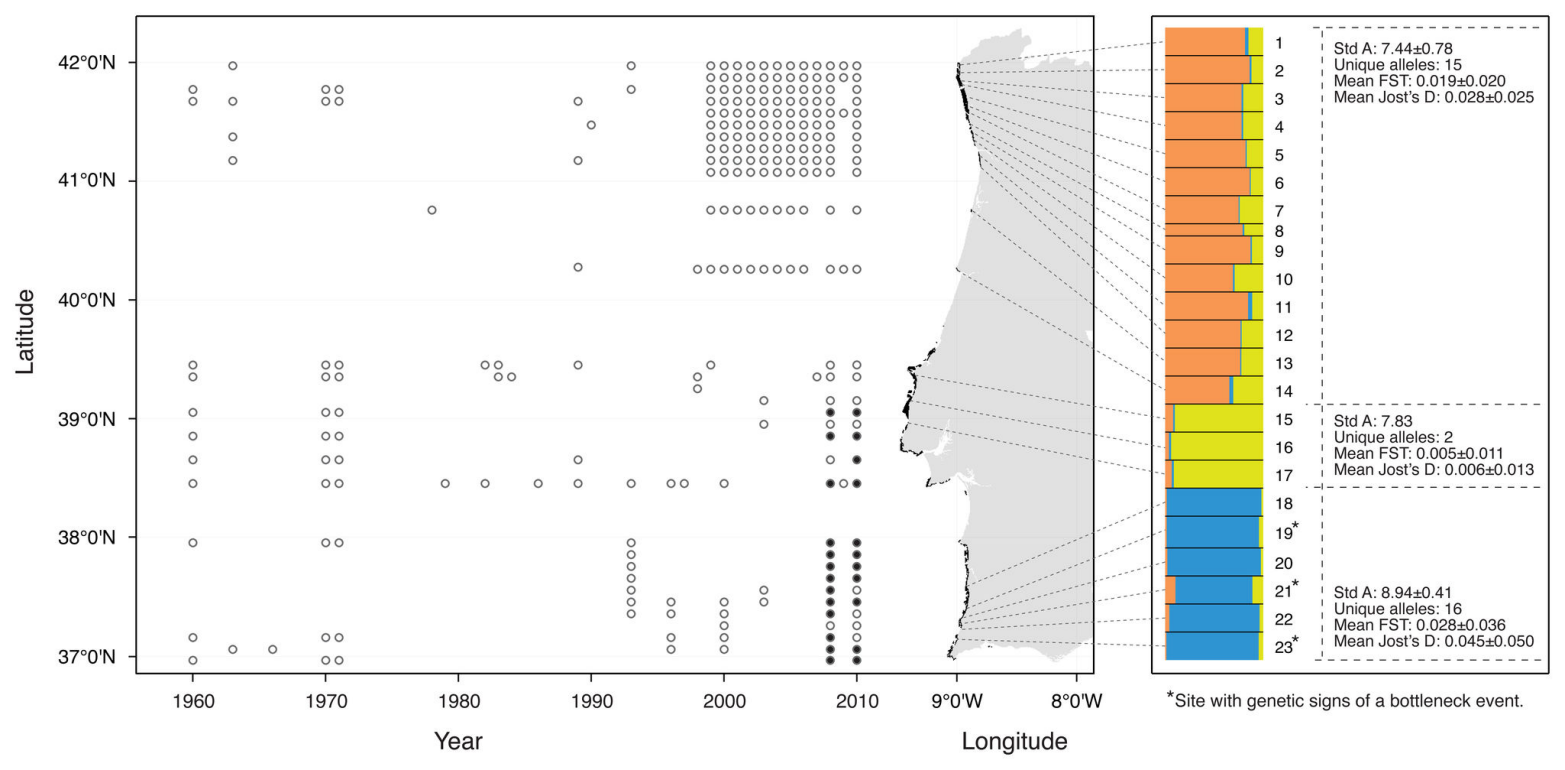
Historical distribution of *S* *. polyschides* based on surveys (2008 and 2010) and literature records (only comparable sites are shown; Open circle: presence, Black circle: absence). Habitat availability shown in black (Rocky reef; data from Portuguese sedimentary charts [70]) along the coast for depths above 20 m (the observed depth distribution of *S* *. polyschides* [92]). Genetic subdivision of *S* *. polyschides* based on STRUCTURE. The proportions of individual multilocus genotypes assigned to K=3 virtual clusters are indicated by the colours. Standardized allele richness (Std A), Mean *F*_ST_, Mean Jost’s D and number of unique alleles per genetic group.

*S* *. polyschides* was well established in the North of Portugal. North of 39^°^ N, populations were present where there was suitable habitat and records were systematic throughout sampling years and literature references (Figure 2). Conversely, south of this latitude, a large decline of *S* *. polyschides* was identified in recent years. Populations of *S* *. polyschides* at most southern sites where it was known to be present were extinct in 2008 and 2010, and the few extent populations were small and variable. Remarkably, at the southern range, some sites that were extinct in 2008 were recolonized from 2008 to 2010.

The density of kelp per sampling site varied between ca. 2 and 26 individuals·m^2^ (Figure 1). The highest densities were registered in the northern sites and a decline was found towards the South (R^2^ = 0.787, p < 0.001). Below the dense northern kelp forests, two sharp declines in density were observed along the coast, the first below latitude 41^°^ N (mean density < 10 individuals/m^2^) followed by an even sparser region in the south (mean density < 5 individuals/m^2^), below latitude 38^°^ N. The among site variation in kelp density, quantified by the coefficient of variation of the densities, was lowest in the north, increasing significantly towards the south (Figure 1; R^2^ = 0.811, p < 0.001).

### Population genetic structure

The Structure analyses, based on both the Evanno [45] and the Pritchard [44] criteria, revealed 3 groups (K=3), separating the northern and the southern sites, plus a central region (Figure 2 Figure S1). When we analysed K=2 (data not shown), the distinct group in the central region appeared as an admixed zone, where alleles from the south and north appeared together (Figure S2). Based on these results, we distinguished a central group and conducted analyses separately for 3 groups, hereafter designated North, Centre and South groups, composed by 14, 3 and 6 sites, respectively.

The genetic differentiation illustrated by the FCA and by the NJ network also revealed differentiation of 3 well supported clusters (Figure S3) corresponding to the same groups determined by the Structure analysis. Moreover, both FCA and NJ network revealed higher genetic distance between sites within the Centre and South than within the North.

**Figure 3:**
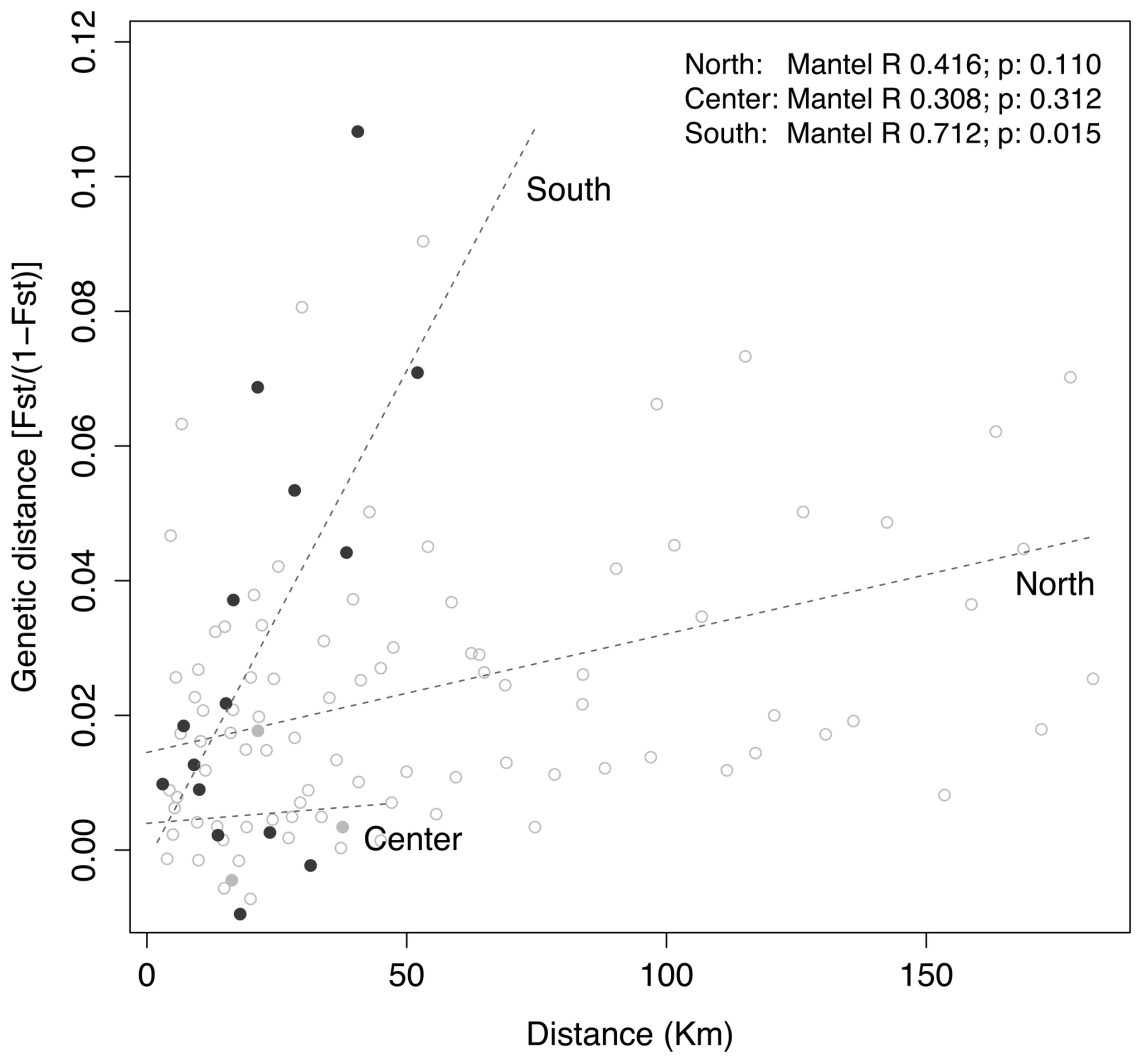
Isolation by distance of *S* *. polyschides* . Estimates of pairwise genetic differentiation (*F*_ST_/(1-*F*_ST_)) plotted against pairwise minimum site distance in kilometres for (i) northern sites (white circles), (ii) central sites (grey circles) and (iii) southern sites (black circles). Mantel non-parametric tests based on 1x10^5^ permutations between pairwise genetic differentiation and pairwise site distance.

Pairwise mean *F*_ST_ and Jost’s D levels of differentiation were higher between the southern sites than between the central or northern sites (Figure 2). These values were significant among sites, among sites within genetic groups and among genetic groups (AMOVA; Table S3). The southern sites followed a model of isolation by distance (Mantel’s R: 0.712, p = 0.015), that was not observed for the North and Center populations (Mantel’s R: 0.416, p = 0.110 and R: 0.308, p = 0.312, respectively) (Figure 3).

### Estimates of genetic diversity

Allelic richness ranged from 3.28 to 7 alleles per site and expected heterozygosity from 0.490 to 0.648. These measures of diversity revealed a significant relation with latitude, increasing towards the south (A: R^2^ = 0.159, p = 0.029; H_E:_ R^2^ = 0.443, p = 0.002; Figure 1). Considering the two main groups, the allelic richness, standardised for 180 individuals within 52.2 km, was 8.94 ± 0,33 for the northern group and 10.62 for the southern. The North showed 17 unique alleles and the South showed 14 unique alleles. When the central admixture zone included, the within group allelic richness, standardised for 90 individuals within 37.6 km was 7.24 ± 0.38 for the North, 7.14 for the Center and 8.94 ± 0.41 for the South. The number of unique alleles was 15 in the North, 2 in the Center, and 14 in the South (Figure 2).

### Inference of population bottleneck

The Wilcoxon test for the null hypothesis of no significant heterozygosity excess across loci showed no signs of bottleneck (Table S4). On the other hand, the M-ratio test retrieved bottleneck signs for three sites located in the Southern region (sites #19, 21 and 23; Figure 2 Table S4). Our survey data show that the forests at sites #19 and #23 were locally extinct in 2008, but recolonized in 2010, the year when our genetic sampling took place.

## Discussion

Our results show persistence of high unique genetic diversity at a species range edge, despite evidence for strong demographic regressions, local extinctions, and extinctions followed by recolonizations. Although we found a decrease in density and an increase in fragmentation with latitude towards the distributional edge of *S* *. polyschides* , the hypothesis of a decrease in genetic diversity with decreasing density was not verified, contradicting expectations. Contrary to density and persistence data, allelic richness and heterozygosity increased towards the more sparsely populated southern range edge. Conversely, marginal southern sites were strongly genetically differentiated, inbreeding coefficients were higher and signs of recent genetic bottlenecks were detected, fitting expectations for small isolated populations undergoing distributional regression. These results raise the question as to why genetic diversity was higher at a low latitude edge despite low population density, fragmentation, genetic isolation, bottlenecks and inbreeding. Below we discuss several potential hypotheses that may explain this pattern.

### Peripheral population decline

A north to south decline in density was evident as a set of latitudinal clines, decreasing density and increasing fragmentation (spatial heterogeneity of the population density). A considerable number of marine species also exhibit latitudinal abundance declines along this coast towards their distributional limits [25]. Southern limits of some cold-water species have shifted north, possibly associated to recent warming associated with the sharp sea surface temperature gradient along this coast [6,63]. However, in the case of *S* *. polyschides* , the decline in density towards the south has been magnified in recent decades, when local populations have been sharply reduced or even disappeared temporally or permanently. As a result, the genetic diversity of southern populations might thus be critically endangered.

### Peripheral population fragmentation

Significant and strong isolation by distance (IBD) was only present at the southern edge region, a likely consequence of habitat fragmentation as seen in other studies [5,63,64]. Moreover, the levels of genetic differentiation between sites were higher and most were significant at the edge. These results show that the northern and central populations are highly connected within the region, whereas those towards the southern periphery of the range have lower gene flow between them, likely due to their occurrence as discrete, geographically isolated patches [16,65]. The observed patterns of abundance can explain this result: *S* *. polyschides* tends to be less dense and more isolated towards the South, sharply increasing genetic distances and consequently IBD in this marginal zone. Such low densities may also increase the variation in mating success, which in turn explains the higher inbreeding values of the southern sites. Low sporophyte densities might be reflected in variable and patchy gametophyte densities, decreasing effective population size as only the spores that happen to settle in close proximity to others (within microscopic scales) will form gametophytes close enough to achieve reproductive success [66,67].

### Phylogeographic influences on diversity

Along the studied range of western Iberia, our results reveal two major genetic groups, North and South, with an admixture region in the Centre. Their high genetic diversity and high number of unique alleles indicate that both regions represent populations that have been large, stable and persistent for long enough to accumulate unique mutations and maintain allelic diversity. They might thus represent genetic groups that were separated at distinct glacial refugia, a role that is also supported by their degree of differentiation from other populations from central Europe (Lamy et al, unpublished data), similarly to other marine species for which the Iberian Peninsula was a glacial refugium [2,5,6,68]. Such reservoirs of unique genetic variation have high conservation value [1,69]. The admixed genotypes in the central region and the rarity of unique alleles there, relative to the northern and southern regions, indicate that this is not an anciently diverged group but rather a more recent contact zone.

The geographical areas between the northern, central and southern forests were sampled and the absence of kelp forests reflected the paucity of suitable rocky habitat. Rocky reefs occur throughout the sampling region, but extensive sandy areas separate these kelp groups (Figure 2 [25,70]). Habitat discontinuity was associated with increased genetic differentiation between patches of the giant kelp *Macrocystispyrifera* in southern California [71] and *Laminaria digitata* in the English Channel [72], which is to be expected given the limits of spore dispersal of such species [72–74]. Such limitations on dispersal are insufficient to assure regular connectivity between the spatially disconnected areas from our study. Yet, despite the breaks in suitable habitat between the genetic groups, some degree of north–south connectivity would be expected from the predominant spring wind and oceanographic circulation along the Portuguese coast [75]. Surface currents could carry floating rafts of *S* *. polyschides* , with high dispersal potential in areas with strong unidirectional currents [76,77]. Such occasional large scale dispersal across km scales must be possible, as it certainly occurred in the past during the colonization of distant available habitat. However, there is strong support for the idea that genetic groups have remained distinct over considerable time, as evidenced by the abundance of alleles unique to the north and south sites. Such genetic boundaries might also be explained by priority colonization effects, which block the spread of later colonizers, as recently proposed for other brown algae [2,78,79].

### Persistence of diversity despite bottlenecks

High genetic diversity is expected where populations have been large and persisted for long periods, without significant effects of drift, local extinctions and bottlenecks. Despite a possible glacial refugial origin of the ancient high and unique southern genetic diversity of *S* *. polyschides* , its recent history of regression and local extinctions recorded along this area was predicted to reflect lower diversity relative to northern Iberia. Conversely, recent bottlenecks and small population size with its associated drift effects, did not noticeably affect diversity patterns along this distributional edge.

How can genetic diversity survive over drastic population size reductions? We hypothesize possible non-exclusive mechanisms that could halt the loss of diversity of such marginal populations. One hypothesis is the occurrence of microscopic stages (such as gametophytes and very young sporophytes) able to persist over unfavourable periods. These could maintain genetic diversity in cryptic stages despite apparent temporary local extinctions and bottlenecks. Experiments on other kelp species, demonstrated that microscopic gametophytes can be maintained in culture for over 7 years [80] and that when growth conditions become favourable, these produce adults faster and more reliably than gametophytes that had never been subject to developmental delay [81,82]. If such long developmental delays also occur in natural field conditions, then even at low densities of adult sporophytes, this delaying strategy may increase effective population size [83], by playing a role analogous to seed banks in plants, allowing temporal persistence of multiple cohorts of potential recruits that store genetic diversity and resume development in favourable years. This hypothesis is however not supported by the evidence from field studies, which identified arrested development stages only on the order of months, not years (e.g., Barradas [84] on this same coast, see also reviews by [81,85,86]). Moreover, a temporal population genetic survey (covering 7–9 years) revealed that a local gametophyte “bank” might not be sufficient to prevent genetic instability of small and isolated populations of the European kelp *Laminaria digitata* [87]. Furthermore, our findings of highest inbreeding coefficients in southern locations do not support the hypothesis of large effective population sizes hidden in cryptic stages.

An alternative hypothesis is the persistence of suitable habitat refugia at southern locations, namely deeper offshore habitats, where light penetration might still be sufficient for kelp persistence, as theoretically predicted for clearer offshore waters [88]. Given the recent increase in sea temperature documented for this transitional zone, hypothetical deep offshore banks functioning as cold water refugia, would provide better niche conditions than shallower warmer coastal sites [88]. Such banks of high evolutionary significance [89] connected to coastal sites [90] could contribute with alleles periodically, thereby halting declines in genetic diversity. Although such deep offshore kelp forests with *S* *. polyschides* exist on underwater mounts (at ca. 40-80 m depths), these are located a few hundred km offshore of the southern distributional edge (Ormonde and Gettysburg Bank [91,92]), and are genetically differentiated from these continental sites (Assis et al. unpublished data), rendering those unlikely to be frequent source populations for this annual species along its continental edge. Moreover, given the strong IBD found in the south, only a network of seamounts could explain the rescuing of diversity of such differentiated and isolated sites.

Local bottlenecks could also be rescued by connectivity from just the few neighbouring remaining patches in the area. Yet, once more, the higher levels of differentiation found between these patches do not support the idea that migration from local remaining sites would be a frequent process. Still, this hypothesis cannot be ruled out since it’s difficult to survey the bottom of the ocean fully and extant populations might occur in areas that we are not aware of. In such a scenario, founder effects could lead to rapid differentiation of patches putatively recolonized by very few occasional migrants from other patches. Yet, this would have been associated with a strong reduction in diversity in such recolonized patches, which is not supported by the presence of many alleles and of alleles that are absent in their neighbours (Figure S2).

A last, but not the least likely hypothesis, is that of shifting genetic baselines, whereby information about the past is lost with increasing extinctions, a problem already reported for other species along this coastline [5,6]. The higher southern diversity does not rule out that strong genetic diversity loss has occurred there. Our bottleneck results are congruent with the hypothesis that, although still richer in genetic diversity than denser northern populations, these southern patches could be the remnants of populations that once had greater genetic diversity.

Extinction of genetic variants is likely to happen frequently without it having been recorded to have ever existed before. This problem calls for studies of the potentially rich and unique genetic diversity that might still exist at pocket range edges. Rear edges below postglacial expansion zones are likely frequent along northern Atlantic shores, and in cases of expansion from introgressed genomes at contact zones, the rear edges may even represent the only surviving populations with the native genomes for the species, as has been reported for other brown algae [93,94]. Marginal populations with such ancient private diversity raise concerns for future climate change predictions, particularly at the warmer edges of the distribution. Besides reporting unique allelic diversity, there is strong need to understand whether local adaptations exist in such endangered populations, increasing their conservation value. Although local adaptations are expected under high selective pressures in genetically distinct populations, their adaptive potential could be constrained in cases where native genetic diversity might have become limiting for their adaptive potential [14].

Our results have clear implications for the conservation of *S* *. polyschides* in particular, in a context of future climate change where bottleneck events may prevail as a result of increasing environmental pressures [95]. In addition to the high conservation value of its genetically diverse and unique peripheral populations, which serve to halt local extinctions [96] and preserve the evolutionary potential of *S* *. polyschides* [14,97], the possible disappearance of these southern populations will also have direct ecological consequences. This kelp species functions as the most important ecosystem engineer of rocky shores along its southern distributional range, forming kelp forests that support a rich community. Thus the loss of kelp forest habitat caused by local extinctions of *S* *. polyschides* negatively affects the diversity and abundance of many associated species.

## Supporting Information

Figure S1 Estimation of the most probable number of groups (K) based on Bayesian clustering for K = 1 to 8 and 25 runs each (STRUCTURE [43]). (A) Mean log-likelihood of the data per K, i e. standard output from Structure. (B) Mean absolute difference of the second order rate of change with respect to K [44].
(PDF)

Figure S2 Allele frequencies for each locus represented by dots of varying diameter. Allele sizes are indicated on the x axis and sites on the y axis. Presence (+) and absence (-) of *S* *. polyschides* per site for the 2008 and 2010 surveys.
(PDF)

Figure S3 Genetic differentiation of *S* *. polyschides* illustrated by a (A) neighbour-joining network of genotypes using Cavalli-Sforza & Edwards [47] pairwise distances. Numbers above the branches are Bayesian posterior probabilities (> 0.50). Inferred groups are divided by dotted lines; and by (B) a Factorial Correspondence Analysis of population multiscores.
(PDF)

Table S1 Number, name, latitude (LAT) and longitude (LON) of site. Records of presence and absence of *S* *. polyschides* for sampling years 2008 and 2010. Mean density records for the 2010 survey.
(XLS)

Table S2 Genetic diversity as allelic richness (A) and Nei’s gene diversity (H_E_), per site and loci. Inbreeding coefficients (*F*_IS_) per site and per loci and deviations from Hardy–Weinberg equilibrium and for linkage disequilibrium between pairs of loci.
(XLS)

Table S3 Pairwise *F*_ST_ between sites. Hierarchical analysis of molecular variance (AMOVA) under 999 permutations with 3 hierarchical levels.
(XLS)

Table S4 Inference of bottleneck for each sampling site using the Wilcoxon test for heterozygosity excess over all loci and M-ratio method. Site number, number of samples per site (n), Wilcoxon test probability (one tail; Wp), Critical MC value, M-ratio (M) and the probability of a smaller M Ratio under equilibrium (Mp).
(XLS)
