# Supplementary material for: High and Distinct Range-Edge Genetic Diversity despite Local Bottlenecks
Source: PLoS One. 2013 Jul 10;8(7):e68646. doi: 10.1371/journal.pone.0068646 (PMC3744244; doi:10.1371/journal.pone.0068646)
Supplement: Figure S1 — (A) Mean log-likelihood of the data per K, i e. standard output from Structure. (B) Mean absolute difference of the second order rate of change with respect to K [44]. (PDF) [file pone.0068646.s001.pdf]

Figure S1. Estimation of the most probable number of groups (K) based on Bayesian clustering for K = 1 to 8 and 25 runs each (STRUCTURE [43]). (A) Mean log-likelihood of the data per K, i.e. standard output from Structure. (B) Mean absolute difference of the second order rate of change with respect to K [44].

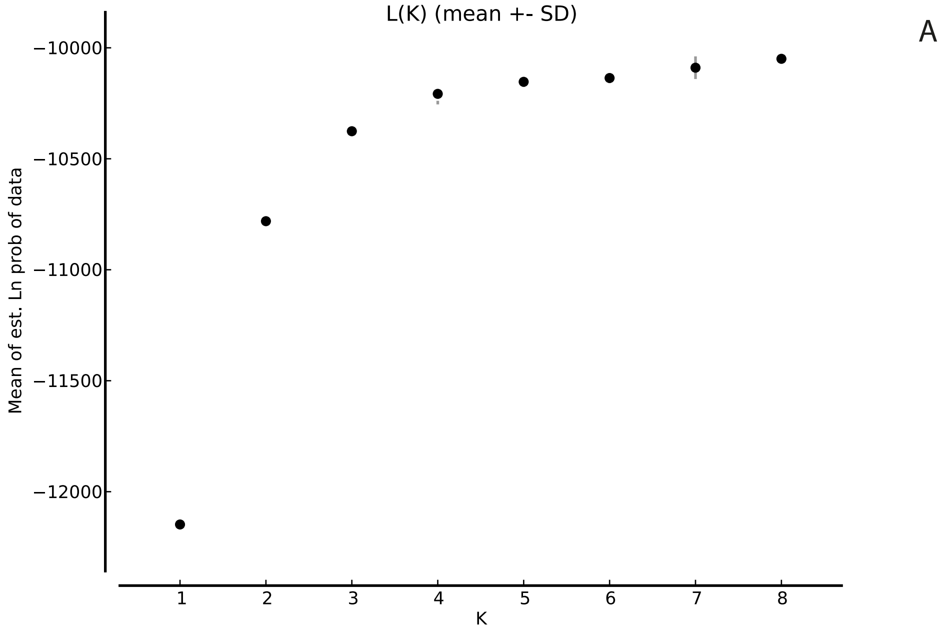

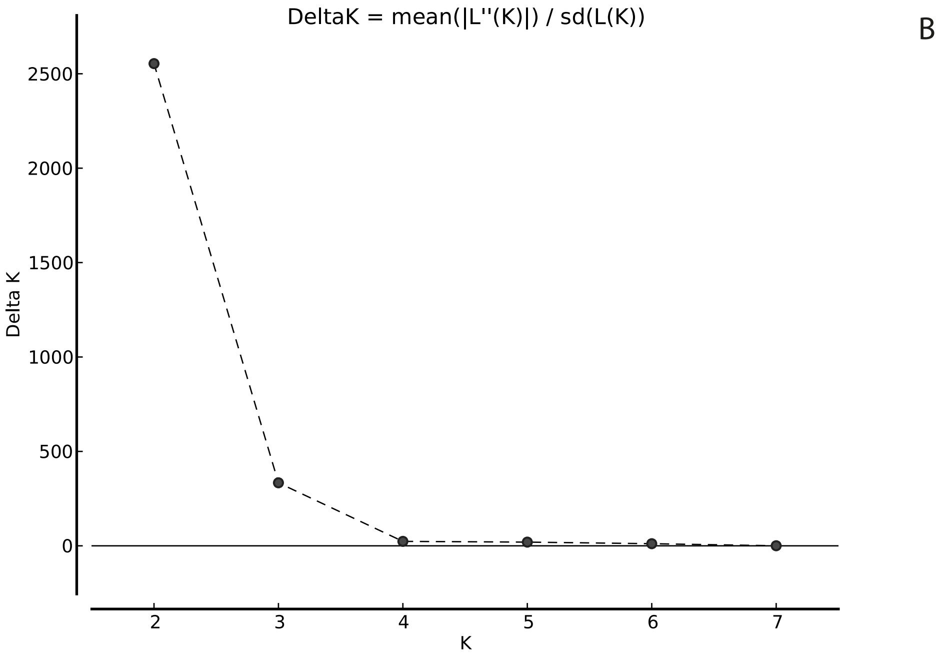

Evanno method output

K	Reps	Mean LnP(K)	Stdev LnP(K)	Ln'(K)	lLn''(K)l	Delta K
1	25	-12147.73333	0.051640	—	—	—
2	25	-10781.18333	0.376386	1366.550000	961.183333	2553.714803
3	25	-10375.81667	0.828050	405.366667	276.350000	333.735919
4	25	-1.02468E+04	2.062038	129.016667	48.600000	23.568918
5	25	-10166.38333	3.218333	80.416667	63.266667	19.658211
6	25	-10149.23333	3.777654	17.150000	42.483333	11.245958
7	25	-1.00896E+04	45.078909	59.633333	19.600000	0.434793
8	25	-10049.56667	5.477834	40.033333	—	—
