# Supplementary material for: High and Distinct Range-Edge Genetic Diversity despite Local Bottlenecks
Source: PLoS One. 2013 Jul 10;8(7):e68646. doi: 10.1371/journal.pone.0068646 (PMC3744244; doi:10.1371/journal.pone.0068646)

Figure S2. Allele frequencies for each locus represented by dots of varying diameter. Allele sizes are indicated on the x axis and sites on the y axis. Presence (+) and absence (-) of S. polyschides per site for the 2008 and 2010 surveys.

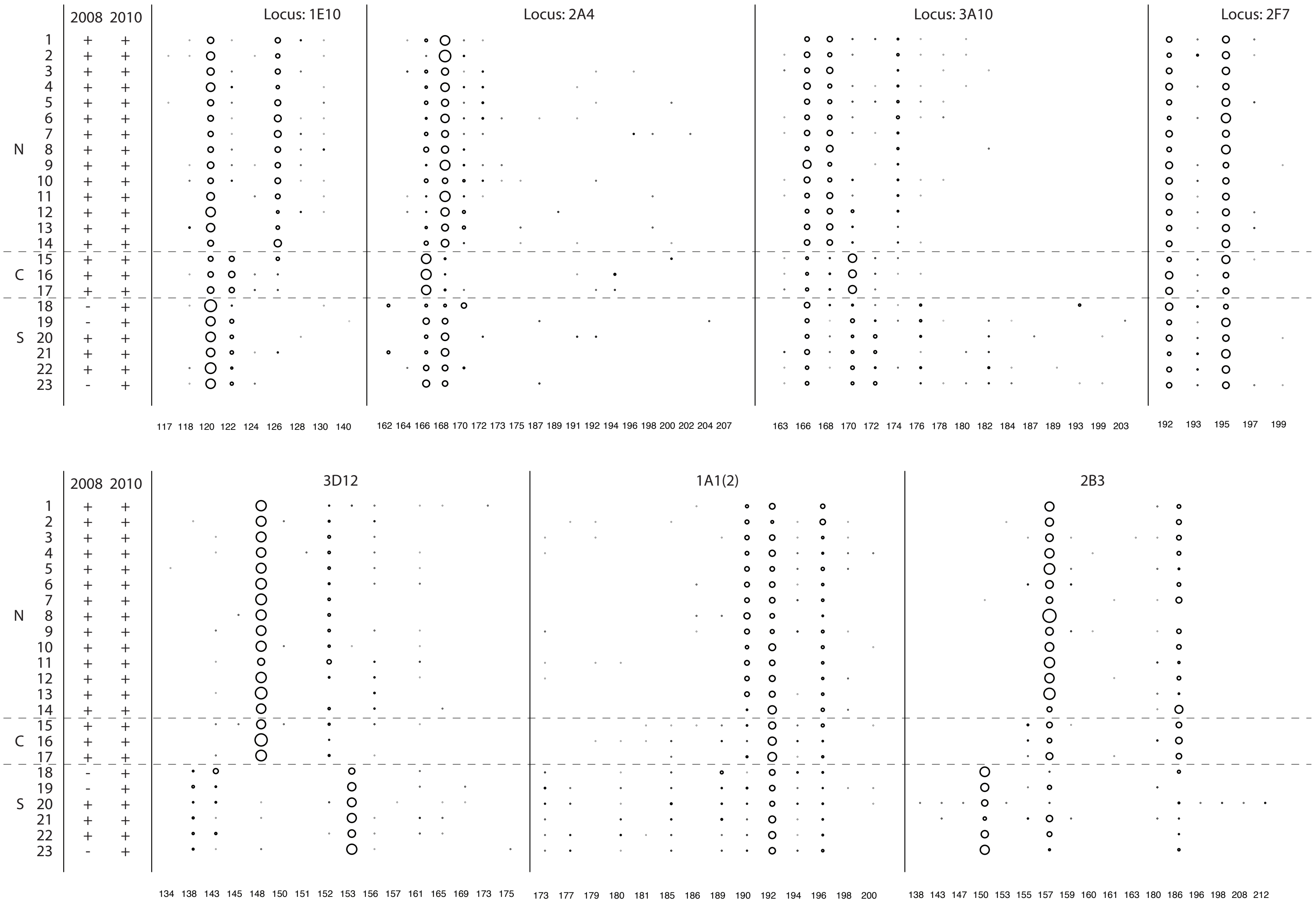

Supplement: Figure S2 — Allele sizes are indicated on the x axis and sites on the y axis. Presence (+) and absence (-) of S . polyschides per site for the 2008 and 2010 surveys. (PDF) [file pone.0068646.s002.pdf]
