# Supplementary material for: High and Distinct Range-Edge Genetic Diversity despite Local Bottlenecks
Source: PLoS One. 2013 Jul 10;8(7):e68646. doi: 10.1371/journal.pone.0068646 (PMC3744244; doi:10.1371/journal.pone.0068646)

Figure S3. Genetic differentiation of S. polyschides ilustrated by a (A) neighbour-joining network of genotypes using Cavalli-Sforza & Edwards [47] pairwise distances. Numbers above the branches are Bayesian posterior probabilities (> 0.50). Inferred groups are divided by dotted lines; and by (B) a Factorial Correspondence Analysis of population multiscores.

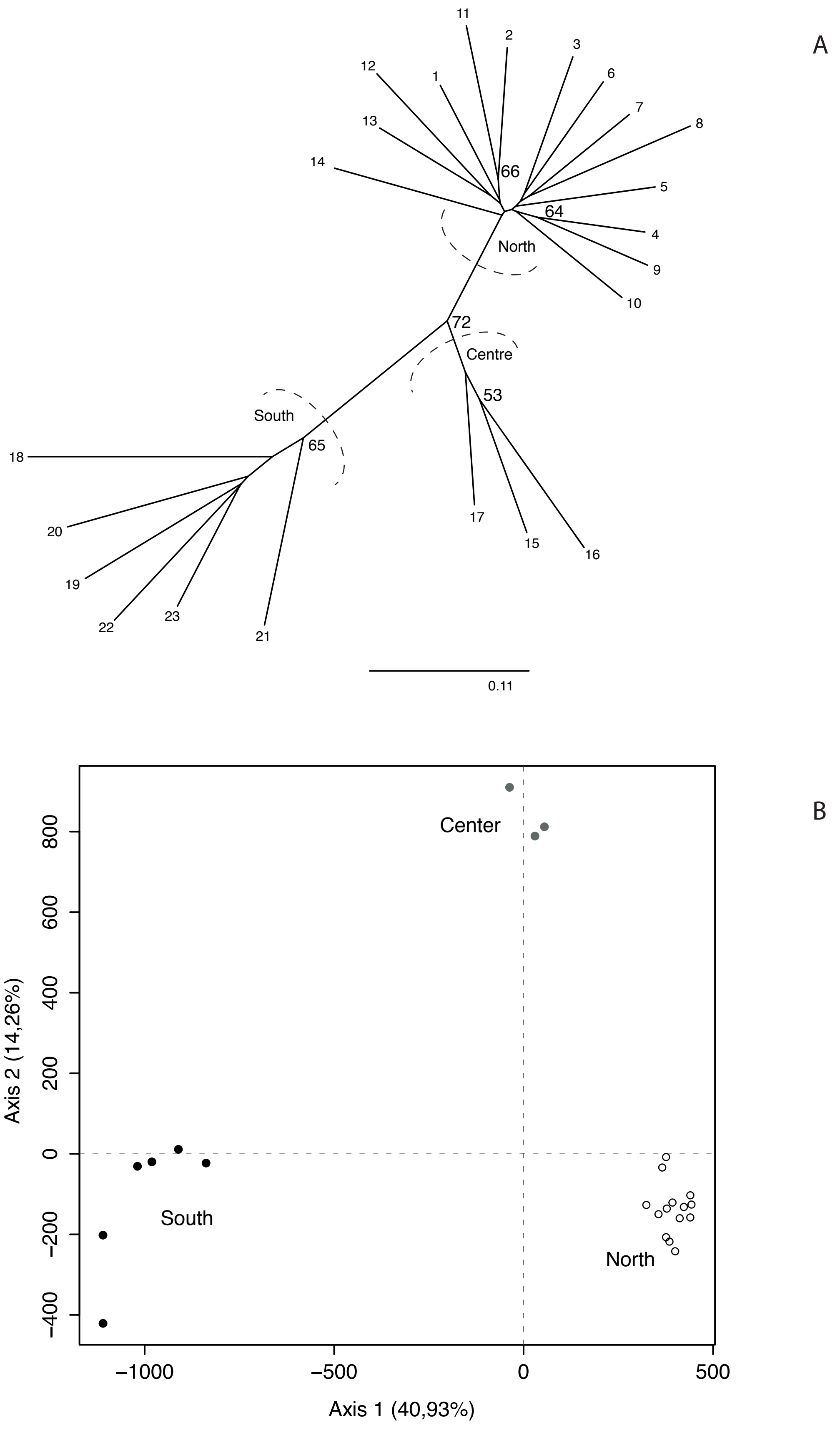

Supplement: Figure S3 — Numbers above the branches are Bayesian posterior probabilities (> 0.50). Inferred groups are divided by dotted lines; and by (B) a Factorial Correspondence Analysis of population multiscores. (PDF) [file pone.0068646.s003.pdf]
